# Variability of Oxaliplatin-Induced Neuropathic Pain Symptoms in Each Cycle and Its Implications on the Management of Colorectal Cancer Patients: A Retrospective Study in South Western Sydney Local Health District Hospitals, Sydney, Australia

**DOI:** 10.1155/2019/4828563

**Published:** 2019-08-01

**Authors:** Endale G. Gebremedhn, Peter J. Shortland, David A. Mahns

**Affiliations:** ^1^School of Medicine, Western Sydney University, Penrith NSW 2751, Sydney, Australia; ^2^School of Science and Health, Western Sydney University, Penrith NSW 2751, Sydney, Australia

## Abstract

Oxaliplatin-induced neuropathic pain limits treatment compliance. However, the variability of neuropathic pain symptoms in each cycle for individual patients and the impacts on treatment compliance remain untested. Data from 322 adult patients who received oxaliplatin-based chemotherapy were extracted based on pattern of chemotherapy, adverse events, and patient survival. Cox regression and survival analyses were employed. Seventy-eight percent of patients developed neuropathic pain that oscillated between a complete absence and presence on a cycle-by-cycle basis. Consequently, the presence of neuropathy in one cycle did not predict the incidence of neuropathy in subsequent cycles. This implies that neuropathic pain need not be a sufficient criterion to reduce, delay, or cease chemotherapy. In the case of multiple system adverse events during combined drug treatment, the responsible cause for dose reduction was not identified. Cox regression analysis revealed that middle age (61–78 years old, *P*=0.003) and oxaliplatin cumulative dose <850 mg/m^2^ (*P*=0.002) were associated with patient mortality. Completion of chemotherapy (8 cycles) and cumulative dose >850 mg/m^2^ of oxaliplatin prolonged the median survival time by 8 and 5 months, respectively. As oxaliplatin-induced neuropathic pain fluctuates across cycles in a manner that varies from patient-to-patient, current assumptions on the predictive nature of the emergence of neuropathy (and its impact on treatment compliance) need to be reconsidered. Detailed patient-by-patient analysis of adverse events should be applied to future studies in order to determine the efficacy of current treatments (and future interventions) and whether neuropathic pain should be retained as a criterion to vary the treatment. Additionally, when two or more system toxicities occurred in cases of combined drug treatment, the causes for drug reduction should be separately recorded.

## 1. Introduction

Oxaliplatin is a third-generation platinum compound that has emerged as a backbone in the treatment of colorectal, gastric, pancreatic, oesophageal, and ovarian cancers both in the adjuvant and metastatic settings [1–3]. However, neuropathy is the major and well-recognized dose-limiting adverse event associated with oxaliplatin treatment. Acute oxaliplatin-induced neuropathic pain develops in 65–98% of patients immediately after the start of infusion and lasts for hours to days [4]. It is characterized by cold-induced dysaesthesias and paraesthesia of the upper extremities and face, cold hypersensitivity, jaw tightness, pharyngolaryngeal dysaesthesia, muscle spasms, fasciculations, and voice changes. Persistent neuropathy becomes more prevalent and affects about 80% of patients [4, 5].

Most clinicians believe that acute neuropathic pain symptoms in the first cycle are transient and resolve within hours to days [4, 6, 7]; yet, a large number of patients continue to experience acute neuropathic pain symptoms in cycle 2 that remains the same in the subsequent cycles [3, 8, 9] with increased risk of developing chronic neuropathy when the cumulative dose exceeds 850 mg/m^2^ [10]. Consistent with this understanding, current clinical treatment guidelines [11] recommend that once severe or persistent neuropathy is observed treatment delay, dose reduction and cessation should be implemented. Intriguingly, numerous trials to prevent chronic neuropathy have failed [12]. Whether this failure arises due to a misunderstanding of the predictive nature of neuropathy in one cycle to the development of persistent neuropathy in successive cycles or relates to the inherent variability of neuropathic pain remains untested.

Better information on the variability and frequency of adverse events and their impact on treatment will enable clinicians to establish strategies that preserve chemotherapy treatment without unduly incurring the development of chronic neuropathy. In this retrospective study, the variability of neuropathic pain symptoms and their impacts on treatment compliance in each cycle was characterized in a cohort of patients from three hospitals in South Western Sydney.

## 2. Materials and Methods

Adult (≥18 years old) colorectal patients who received oxaliplatin-based chemotherapy at three South Western Sydney Local Health District Hospitals (Liverpool, Campbelltown & Bankstown) from 2011 to 2015 were included this retrospective study. Ethical approval was obtained from the NSW Human Research and Ethics Committee (HE17/113).

Data were retrieved from a single database of the three hospitals for five consecutive years (2011–2015) and information was extracted on the following: (1) baseline characteristics (age, gender, patient hospital identification number, diagnosis, cancer stage, admission criteria, etc.); (2) patterns of care (treatment regimen and starting and cycle-by-cycle dose (cumulative dose >850 mg/m^2^ claimed to cause chronic neuropathy [13]), treatment cycle, dose reduction, and laboratory investigation values); (3) frequency and magnitude of acute and chronic neuropathies (paraesthesia, dysaesthesia, cold triggered pain, and motor deficits), other adverse events (Table 1, gastrointestinal, haematological, and renal side effects), number of patients who received a dose reduction (and the extent to which this is attributable to neuropathy) and dropout rates (number of patients who completed the entire 8 treatment cycles), and (4) survival rates of treated cancer patients. Neuropathic pain symptoms and haematological and other adverse events (AE) were graded using the National Cancer Institute Common Terminology Criteria for Adverse Events (NCI CTC AE version 4.0) (Table 1) [14, 15]. Oxaliplatin-induced neuropathy was scored as Grade 1 neuropathic pain (mild: not interfering with function), grade 2 (moderate: interfering with function but not interfering with activity of daily life), grade 3 (severe: interfering with activity of daily life), and grade 4 (disabling: impairs function) [15].

### 2.1. Statistical Analysis

Descriptive statistics was employed to determine the prevalence of neuropathy and cumulative dose that caused both acute and chronic neuropathies. The correlation between the cumulative dose and the severity of both acute and chronic oxaliplatin-induced neuropathies were examined using Spearman or Pearson correlations as appropriate. Postchemotherapy survival of patients was determined using a survival analysis model (survival curve/life table). The time when death occurred after completing chemotherapy was also determined. Time to the occurrence of death was measured in months from the completion of chemotherapy to the occurrence of death. The factors associated with adverse events were determined using proportional Cox regression analysis model, and the strength of association between dependent and independent variables was assessed using odds ratio (OR).

## 3. Results

### 3.1. Characteristics of Study Subjects and Chemotherapy Profiles

Three-hundred and twenty-two colorectal cancer patients treated with oxaliplatin-based chemotherapy between 2011 and 2015 were included in the study. The majority of patients had adjuvant (no metastasis) colorectal cancer, with most patients being treated with FOLFOX chemotherapy regimen (Table 2). In patients with metastatic colorectal cancer, 75% were treated with FOLFOX (folinic acid, fluoruracil, and oxaliplatin) and 23% with XELOX (oxaliplatin and capecitabine) (Table 2).

### 3.2. Incidence of Oxaliplatin-Based Chemotherapy Adverse Effects

**Figure 1:**
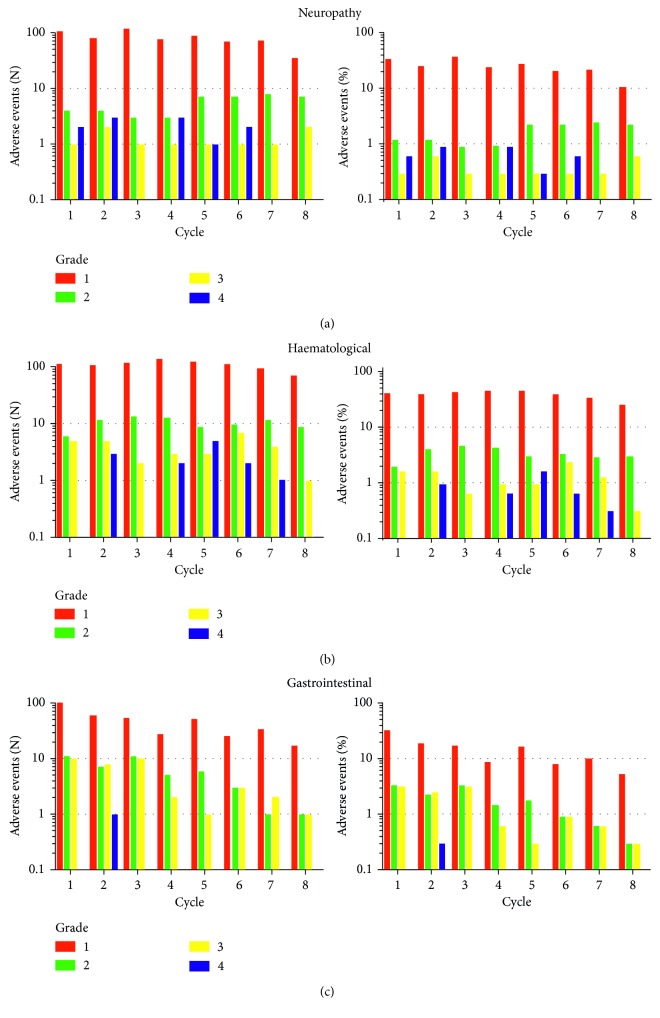
The number (left panels, *N*) and incidence (right panels, %) of patients who developed adverse events leading to a change in treatment including peripheral neuropathy (top row, *N* = 250), haematological toxicity (middle row, *N* = 267), and gastrointestinal toxicity (bottom row, *N* = 229).

The cumulative dose of oxaliplatin that patients received varied between 110 and 2160 mg/m^2^. For FOLFOX treatments, chemotherapy cycles were spaced every 2 weeks, whereas XELOX cycles were spaced every three weeks for up to 8 cycles. The overall incidence of patients developing at least one episode of neuropathy, haematological, gastrointestinal, or renal toxicities was 78%, 83%, 71%, and 3%, respectively. Cycle-by-cycle incidence of all adverse events varied across cycles. For example, the maximum incidence of neuropathy from any of the cycles was 36%, whereas the overall incidence during the course of chemotherapy was 78%, indicating that individual patients need not experience cycle-by-cycle neuropathy that is sustained throughout the course of chemotherapy. The variability of overall per cycle incidence of adverse effects was also true for haematological and gastrointestinal adverse events (Figure 1).

As the completeness with which adverse events were documented on a cycle-by-cycle basis in the database varied between neuropathy (97%), gastrointestinal (95%), renal (15–45%), and haematological (45–65%) events, subsequent subject-by-subject analyses focused on the well-documented (97%) high-incidence (>78%) neuropathy events. The breakdown of neuropathy incidence by sex, treatment regimen, age, previous chemotherapy, and cancer types is shown in Table 3.

**Figure 2:**
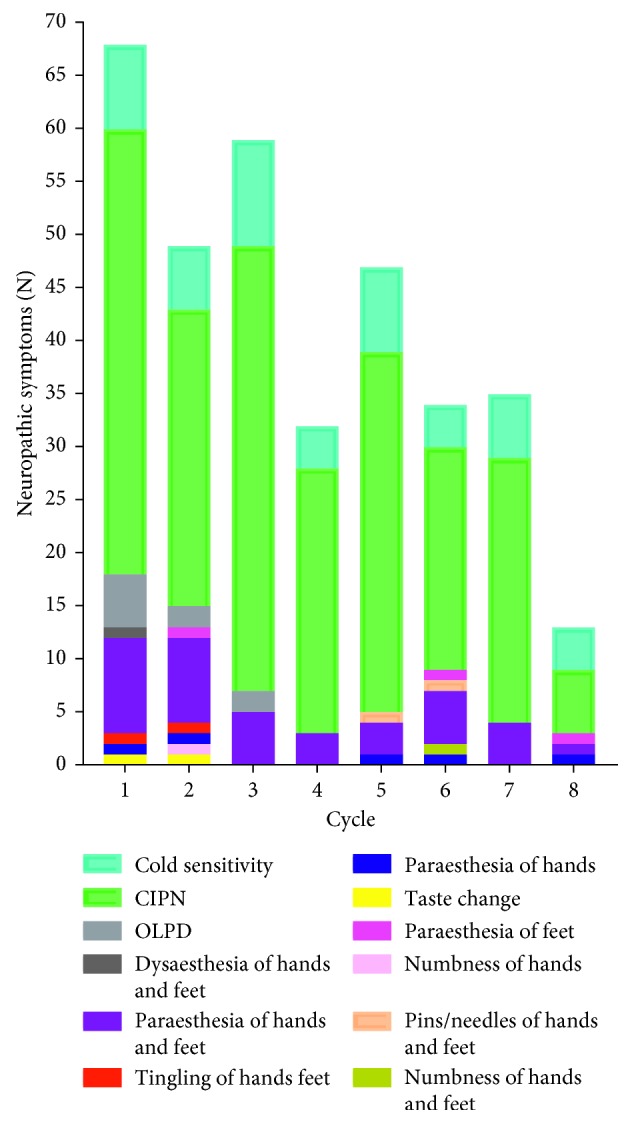
Documented incidence of neuropathic pain and specific types of symptoms induced across cycles. CINP: cold-induced neuropathic pain; OLPD: orolaryngopharyngeal dysaesthesia. In the rest of patients, it was not specified whether neuropathic pain was triggered by cold or not.

Cold hypersensitivity is a commonly reported problem in patients who received oxaliplatin-based chemotherapy. On those occasions where the characteristics of oxaliplatin-induced neuropathic pain were documented, 74% of the accounts noted cold sensitivity and CINP across all cycles (Figure 2), whereas in the remainder of patients, the presence of a neuropathy (grade ≥ 1) was noted but its characteristics were not specified. The types of neuropathic symptoms are summarized in Figure 2.

### 3.3. Variability of Oxaliplatin-Induced Neuropathic Pain and Its Implications

**Figure 3:**
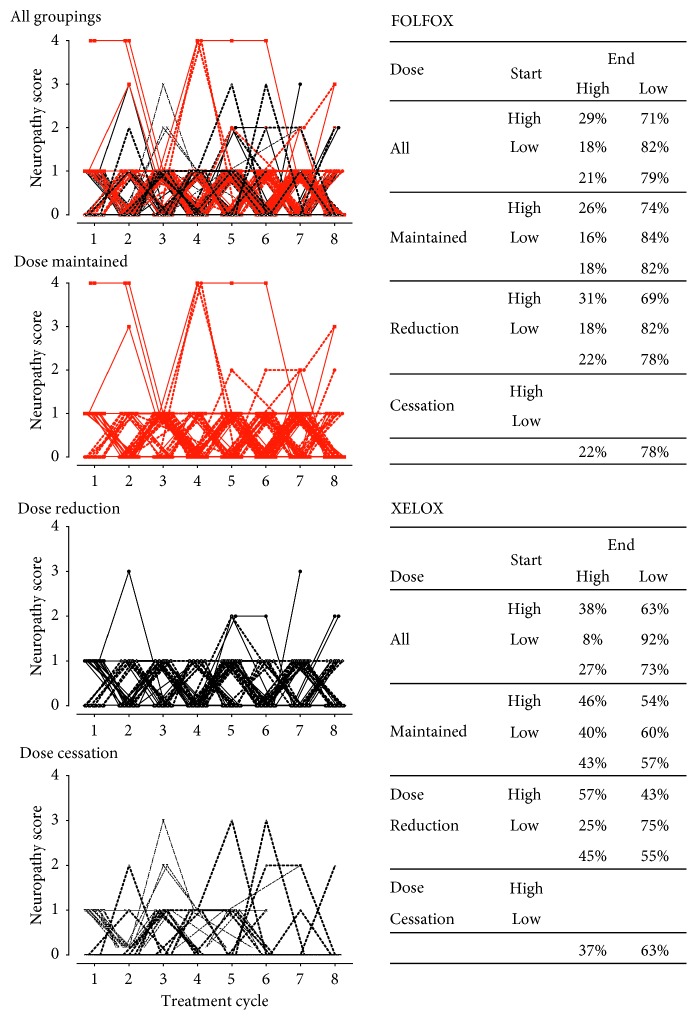
Cycle-by-cycle variance in neuropathic pain for individual patients on FOXFOX and XELOX treatment regimens. In the left hand panels subject-by-subject neuropathy scores for those receiving FOLFOX were plotted and categorized based on the presence (≥1, solid line) and absence (<1 dotted line) of neuropathy in cycle 1 (*n* = 200). In the lower 3 panels, the data were grouped based on whether the FOLFOX regimen was maintained (*n* = 81), reduced (*n* = 57), or ceased (*n* = 49). The right hand panel tabulates the impact of the presence or absence of neuropathy in cycle one on the neuropathy scores at cycle 8 (end) of FOLFOX and XELOX treatments. The presence of patient-by-patient oscillations in the neuropathic score indicates that the emergence of neuropathy in one cycle is not predictive of the developing a higher score in the subsequent cycles. This observation held true across FOLFOX and XELOX even when regimens were subject to dose reduction and/or cessation indicating that neuropathic pain alone is not a sufficient reason to cease treatment.

In Figure 3, the FOLFOX data (*n* = 157) were replotted to show the cycle-by-cycle severity (grade) of neuropathy experienced on a patient-by-patient basis. In the uppermost panel, the data were categorized according to whether there was absence (<1, dotted lines) or presence (≥1, solid lines) of neuropathy in cycle 1. Plotting the data in this categorized manner revealed that the presence (≥1) and absence (=0) of neuropathy varied from cycle to cycle. In the lower panels, the same data were grouped according to whether the treatment regimen was maintained, reduced, or ceased over 8 cycles of treatment, highlighting that neuropathy did oscillate from cycle-to-cycle on a subject-by-subject basis within all three groupings. In the tables accompanying Figure 3, the effect of the presence (or absence) in cycle one on the presence of neuropathy at cycle 8 was tracked on patient-by-patient basis. As only 3 patients in the cessation group continued beyond cycle 6, it was not possible to conduct comparable analysis in this group. When all groups were included, this analysis revealed that 71–82% of patients end their treatment with no reported neuropathy, regardless of whether neuropathy was present or absent in cycle 1. This bias towards the absence of neuropathy in cycle 8 was repeated across all subgroupings (maintained and dose reduction) in the FOLFOX and XELOX regimens. Consistent with this analysis, the positive correlation between cumulative dose >850 mg/m^2^ (*r* = 0.212, *P*=0.037) and neuropathy grades was only observed at cycle 5. This implies that on a patient-by-patient basis, the emergence of neuropathy in one cycle need not predict the presence of ongoing or subsequent neuropathy. Importantly, a similar oscillating pattern was observed in those patients where the dose of oxaliplatin was maintained throughout the treatment period (Figure 3). According to the literature, grade 1 neuropathy is considered to be mild and does not have an effect on treatment compliance [15]. However, in the current study, grade 1 neuropathic pain was the main cause for dose reduction in the majority of patients across all cycles (Figure 3).

### 3.4. Impact of Oxaliplatin-Induced Neuropathic Pain and Other Chemotherapy Adverse Effects on Treatment Compliance

**Figure 4:**
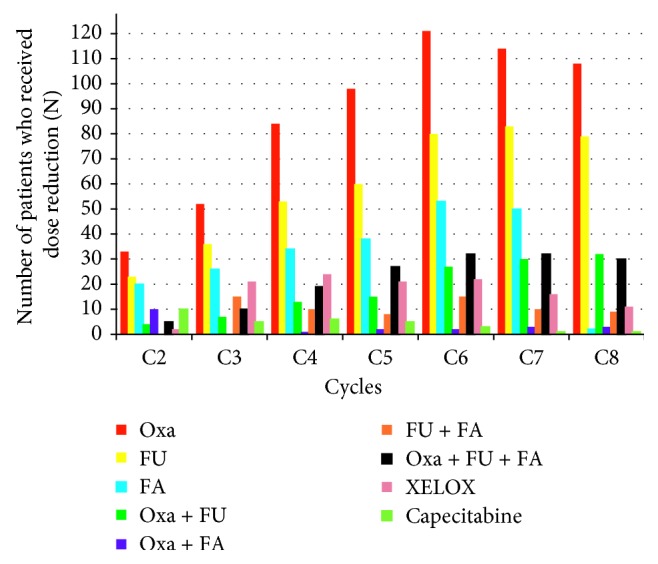
Number of patients who received dose reduction for a single drug or combinations of drugs in each cycle irrespective of the type of treatment regimens. Fluorouracil (FU); folinic acid (FA); XELOX: oxaliplatin + capecitabine.

The type and magnitude of dose reduction of anticancer drugs could affect the quality of treatment outcome, and this has not been well addressed in the clinical research literature. In this retrospective study, a large number of patients received a dose reduction for oxaliplatin (10–38%) as a single drug, followed by fluorouracil (7–26%) and folinic acid (0.6–16%), respectively, across the chemotherapy cycles. In addition, a large number of patients received a dose reduction for oxaliplatin plus fluorouracil and FOLFOX, respectively, as the chemotherapy cycles are increased (Figure 4).

**Figure 5:**
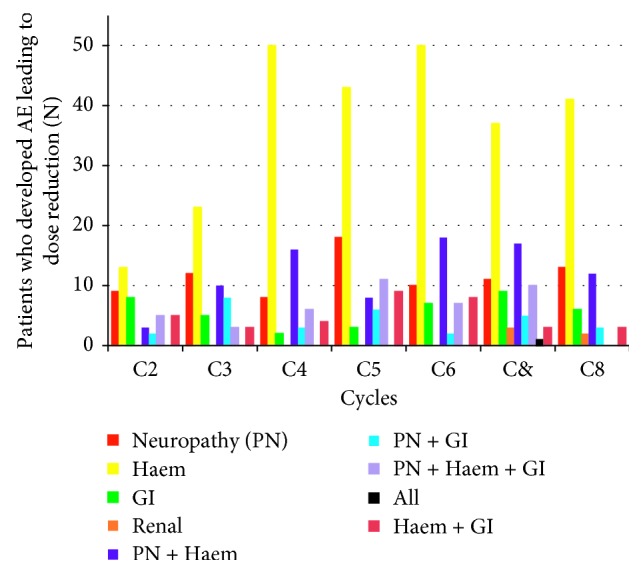
Documented causes for chemotherapy dose reduction and the number of patients who needed dose reduction in each cycle. aE: adverse events; Haem: haematological; GI: gastrointestinal. These data are only for the patients where the cause for dose reduction was clearly reported. There were a large number of cases where the reasons for dose reduction were not reported.

In contrast to the previous reports [4, 16], haematological toxicity was the main adverse advent recorded for dose reduction, followed by neuropathy plus haematological toxicity and neuropathy alone, respectively, across cycles (Figure 5). Neuropathy along with any other adverse events resulted in dose reduction in 37-51% of the patients in any given cycle. However, for a large proportion of patients, the cause for dose reduction was not documented in the database (Figure 5). Across cycles, only 0.3% to 1.8% of patients were subjected to treatment delay and 0.9% to 2.2% of patients ceased the treatment due to chemotherapy-induced adverse events.

### 3.5. Patient Survival Analysis after Chemotherapy and Associated Factors

**Figure 6:**
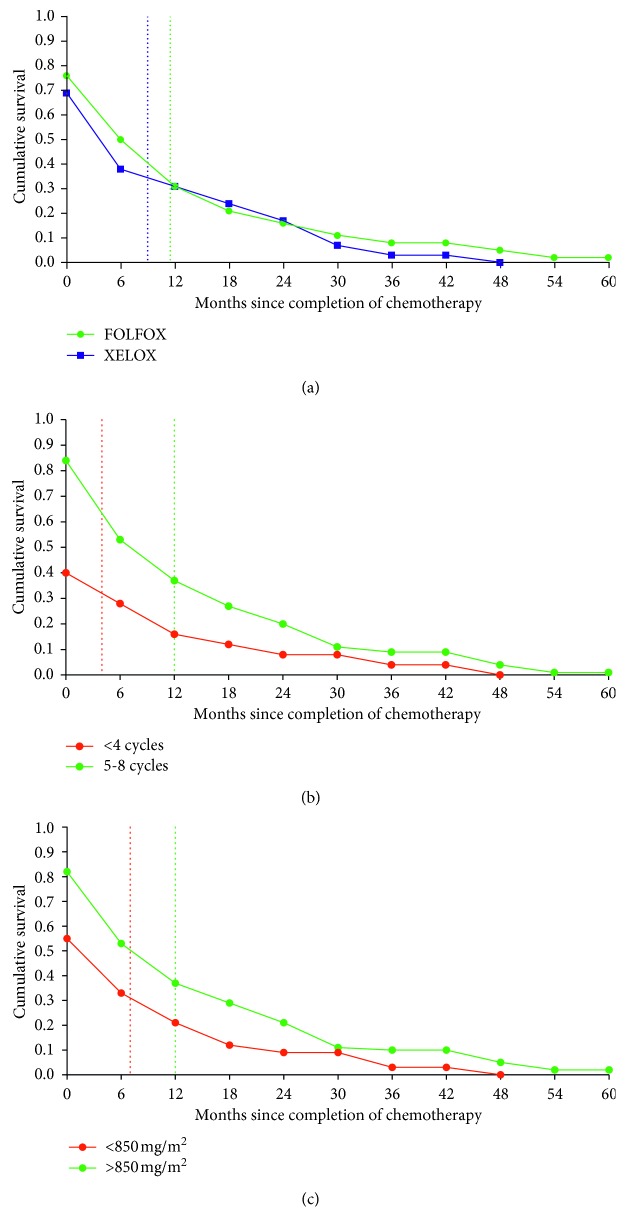
Cumulative probability of patient survival by (a) regimen, (b) cycles covered, and (c) cumulative dose received in the South Western Sydney between 2011 and 2015. Patients who received FOLFOX survived longer compared to XELOX (median value: 11.5 months vs. 9 months; *n* = 211 vs. *n* = 105, respectively). The vertical dotted lines indicate median survival time for each group.

From the Cox regression analysis, age (61–78 years old) (AOR = 3.005; 95% CI = 1.466–6.162, *P*=0.003) and oxaliplatin cumulative dose <850 mg/m^2^ (AOR = 2.066; 95% CI = 1.313–3.314; *P*=0.002), respectively, had a strong association with patient mortality after chemotherapy. The survival analysis shows that patients who received FOLFOX treatment regimens survived up to 2.5 months longer (median values: 11.5 months versus 9 months) compared to XELOX (Figure 6(a)). However, in this study, patients who received 5–8 cycles of chemotherapy had a longer survival times with a median of 12 months compared to those who received ≤4 cycles (4 months, Figure 6(b)). Moreover, patients who received a cumulative dose of oxaliplatin of >850 mg/m^2^ had a longer survival time (median values: 12 months) compared to those that received <850 mg/m^2^ (median values: 7 months, Figure 6(c)). Collectively, these results support the increased benefit of prolonged treatment.

There was no significant difference in patient survival based on gender and cancer stage (nonmetastatic and metastatic cancer). However, middle-aged patients (51–60 years old) had a relatively longer survival time compared to either younger (30–50 years old) and/or older patient groups (61–78 years old) with a median survival time of 24 months.

## 4. Discussion

Oxaliplatin is a common anticancer drug for the management of colorectal cancer and other cancers such as gastric, pancreatic, ovarian, and testicular cancers [10]. According to the literature, the commonest dose-limiting side effect of oxaliplatin is neuropathic pain which can occur within hours of infusion of the drug [4]. However, none of the clinical trials which have been conducted to date have found an effective prophylactic or treatment drug for oxaliplatin-induced neuropathic pain [12].

The overall incidence of oxaliplatin-induced neuropathic pain reported in this study (78%) was consistent with the earlier studies [4, 17, 18]. The patient-by-patient analysis in this study revealed that neuropathic pain was not consistent throughout the treatment cycles but oscillated from present to absent on a cycle by cycle basis. For those patients that display neuropathy in cycle one, this is consistent with the published literature, where oxaliplatin-induced neuropathic pain is reported to be high in the first cycle and transient and resolved within few days [4, 8, 12]. Furthermore, in this study, following the first incidence of painful neuropathy (be it in the first or second cycle), all patients displayed a resolution, before neuropathy re-emerges in subsequent cycles. The presence of oscillating pain is in marked contrast to the prevailing view that once neuropathy is initiated, subsequent doses of oxaliplatin induce persistent neuropathy [3, 8, 9]. The mismatch between the conventional dogma and our results could result from either a lack of systematic reporting of neuropathic pain symptoms or the reliance on aggregated (average) data where the responses of individual are lost from the analysis. This latter point was evident when we categorized individual responses into two groups, i.e., those with/without high neuropathy in the first cycle and documented that both categories progressed to cycle-by-cycle oscillations (Figure 3) vis-à-vis the same data presented in an aggregated form (Figure 1) that showed a persistent neuropathy in up to 36 % of patients, with the contribution of individual patients varying form cycle-by-cycle. Collectively, these results question whether observing neuropathy in any given individual is a sufficient reason to cease treatment and whether cycle-by-cycle analyses that preserve the individual responses should be evaluated in all retrospective analysis and future clinical trials [8, 12]. For example, in this study, grade 1 neuropathic pain was the most frequently recorded adverse event associated with dose reduction, but it was not correlated with, or predictive of, a high neuropathy score at cycle 8.

It is well-documented that combinations of anticancer drugs can improve treatment efficacy [12, 19]. However, there was no clear evidence as to why oxaliplatin alone has been the focus of claims for neuropathy, especially when two or more drugs were given in combination with oxaliplatin. A previous study showed that the addition of the platinum-derivative anti-cancer drugs to the combination of fluorouracil and leucovorin increased the rate of patients developing chronic neuropathy [2]. Moreover, it is not explained in the literature why, and to what extent [4], the doses of other combined anti-cancer drugs need to be reduced when the patients develop neuropathy. The absence of detailed reporting of the specific types of adverse effects of each drug produced and the measures taken for single or combined drugs make patient management and treatment outcome poor as shown in our previous study [16]. Furthermore, in the current study, haematological toxicity was the main criteria for dose reduction, followed by neuropathy and gastrointestinal adverse effects [19]. Previous animal and human studies have reported that haematological toxicity is mainly caused by fluorouracil (1–6%) and not by oxaliplatin [17, 18, 20] with a 1-2% increase in the risk of developing chronic neuropathy with combined treatments [2, 18]. However, there was no clear report in this database, or the previously published literature, as to which systemic adverse effects were responsible for dose reductions when the patients developed two or more systemic toxicities in each cycle for patients who received combined drug treatment. Moreover, there was no evidence in the database and published literature whether pain which was reported by the patients was caused by neuropathy alone and/or was also contributed by haematological, gastrointestinal, and renal problems.

From the proportional Cox regression analysis, age (61–78 years old) and oxaliplatin cumulative dose (<850 mg/m^2^) had the strongest positive associations with patient mortality after chemotherapy. The presence of increased survival times in middle-aged patients (51–60 years old) compared to either younger (30–50 years old) or older patient groups (61–78 years old) is well aligned with the current Australian bowel cancer (including colorectal cancer) screening strategies that targets patients aged over 50, [21–24] suggesting that significant benefits may be derived by dropping the commencement of screening to 40 and, thereby, initiating treatment prior to the progression to high-grade disease and metastases [21–24]. Additionally, patients who received FOLFOX treatment had longer survival times, followed by XELOX and oxaliplatin monotherapy (11.5 vs 9 vs 4.5 months, respectively). Survival analysis (by factor) confirmed that patients who completed 8 cycles of chemotherapy and those who received >850 mg/m^2^ cumulative dose of oxaliplatin had longer survival times, indicating that delay or cessation leads to poorer treatment outcome as reported in this and prior studies [12, 16]. Moreover, the IDEA clinical trial study failed to confirm overall noninferiority of 3 vs. 6 months treatment of XELOX and FOLFOX regimens but did show inferiority of shorter duration FOLFOX treatment in high risk subgroups [25–27]. In our study, patients were not stratified based on tumor stage; however, when patient survival was compared between adjuvant and metastatic cancer patients, we did not observe a statistical significance difference. The need to apply the findings of the IDEA study with caution is emphasised by the observations that low-risk patients with pericolonic tumor implants and extranodal disease extension have a higher risk of disease relapse [25, 27, 28]. The different outcomes from the previous studies could be due to variations in the type of regimens used (FOLFOX vs. XELOX, etc.) for adjuvant and neoadjuvant (chemoradiotherapy) therapy, duration of therapy, and dosage in an attempt to balance between patient treatment compliance and achieving disease-free survival [18, 20, 27–33]. This implies that further well-designed and controlled studies are needed to determine whether 8 or less cycles of chemotherapy and FOLFOX or other regimen is effective to prolong the life in patients with colorectal cancer.

## 5. Conclusions

Individual patients experience oxaliplatin-induced neuropathic pain that fluctuates across cycles, highlighting the need to base clinical judgments about treatment compliance on the individual's response rather than relying on assumptions based on the aggregated population response. When two or more toxicities occurred in combined drug treatment, the causes for any drug reduction due to adverse events should be separately recorded. Importantly, the current belief that neuropathy is the predominant causal factor affecting treatment compliance should be revisited. In this study, patients who received 8 cycles of chemotherapy had a longer survival time compared to those did not.

## Figures and Tables

**Table 1:** Scoring criteria used for chemotherapy-induced adverse events.

CTCAE grading		0	1	2	3	4
Neurotoxicity	Peripheral	None	Loss of tendon reflexes or paraesthesia (including tingling) but not interfering with function	Sensory alteration or paraesthesia (including tingling) interfering with function but not interfering with daily living	Interfering with daily living	Disabling
	Cold-induced perioral paraesthesia	None	Mild	Moderate	Severe	Disabling
	Cold-induced pharyngolaryngeal dysesthesia	None	Mild	Moderate	Severe	Disabling
	Cold-induced paraesthesia in the upper extremities	None	Mild	Moderate	Severe	Disabling
	Cold-induced paraesthesia in the lower extremities	None	Mild	Moderate	Severe	Disabling

GI toxicity (hepatic)	Diarrhoea	None	Transient <2 days	Tolerable but >2 days	Intolerable requiring therapy	Haemorrhagic dehydration
	Nausea/vomiting	None	Nausea	Transient vomiting	Vomiting requiring therapy	Intractable vomiting
	Constipation	None	Mild	Moderate	Abdominal distention	Distention and vomiting
	Oral (stomatitis)	None	Soreness/erythema	Erythema, ulcers, can eat solids	Ulcers, requires liquid diet only	Alimentation not possible
	Bilirubin	≤1.25 × *N*	1.26–2.5 × *N*	2.6–5 × *N*	5.1–10 × *N*	>10 × *N*
	SGOT/SGPT	≤1.25 × *N*	1.26–2.5 × *N*	2.6–5 × *N*	5.1–10 × *N*	>I0 × *N*
	Alkaline phosphatase	≤1.25 × *N*	1.26–2.5 × *N*	2.6–5 × *N*	5.1–10 × *N*	>10 × *N*

Haematological toxicity	Haemoglobin (g/100 ml)	≥11.0	9.5–10.9	8.0–9.4	6.5–7.9	<6.5
	Leukocytes (1000/cmm)	≥4.0	3.0–3.9	2.0–2.9	1.0–1.9	<1.0
	Granulocytes (1000/cmm)	≥2.0	1.5–1.9	1.0–1.4	0.5–0.9	<0.5
	Platelets (I000/cmm)	≥100	75–99	50–74	25–49	<25
	Haemorrhage	None	Petechiae	Mild blood loss	Gross blood loss	Debilitating blood loss

Renal toxicity	BUN or blood urea	≤1.25 × *N*	1.26–2.5 × *N*	2.6–5 × *N*	5–10 × *N*	>I0 × *N*
	Creatinine	≤1.25 × *N*	1.26–2.5 × *N*	2.6–5 x *N*	5–10 × *N*	>I0 × *N*
	Proteinuria	None	I+, <0.3 g/100 ml	2-3+, 0.3–1.0 g/100 ml	4+, >1.0 g/100 ml	Nephrotic syndrome
	Heamaturia	None	Microscopic	Gross	Gross + clots	Obstructive uropathy

*N* = upper limit of the normal value; BUN = blood urea nitrogen; SGOT = serum glutamic-oxaloacetic transaminase; SGPT = serum glutamic-pyruvic transaminase; CTCAE = Common Terminology Criteria for Adverse Events.

**Table 2:** Baseline characteristics of study subjects.

Variable	Frequency (*n*)	Percentage (%)
Type of colorectal cancer		
Adenocarcinoma of caecum	61	18.9
Adenocarcinoma of ascending colon	30	9.3
Adenocarcinoma of transverse colon	21	6.5
Adenocarcinoma of descending colon	10	3.1
Adenocarcinoma of sigmoid colon	109	33.9
Adenocarcinoma of rectosigmoid junction	28	8.7
Adenocarcinoma of rectum	63	19.6

Starting dose of oxaliplatin (IV) in the treatment regimen		
85 mg/m^2^	214	66.5
100 mg/m^2^	5	1.6
130 mg/m^2^	103	32

Starting dose of fluorouracil in the treatment regimen		
400 mg/m^2^ (IV) plus 2400 mg/m^2^ (CIVP)	211	65.5
NA	111	34.5

Starting dose of folinic acid (FA) in the treatment regimen		
50 mg/m^2^	1	0.3
200 mg/m^2^	207	64.3
400 mg/m^2^	3	0.9
NA	111	34.5

Treatment regimen	Adjuvant: *n* (%)	Metastatic: *n* (%)
FOLFOX	144 (44.7)	67 (20.8)
XELOX	84 (26.1)	21 (6.5)
FOLFOX + avastin	1 (0.3)	0 (0)
Oxaliplatin alone	4 (1.2)	1 (0.3)

NA: not applicable; CIVP: central intravenous pump; IV=intravenous.

**Table 3:** Factors related to the incidence of peripheral neuropathy.

Factor	Peripheral neuropathy, *N* (%)		
	Yes	No	Not documented
Sex			
Male	141 (43.8)	40 (12.4)	3 (0.9)
Female	109 (33.8)	27 (8.4)	2 (0.6)
Treatment regimen			
FOLFOX	157 (48.8)	49 (15.2)	5 (1.5)
XELOX	89 (27.6)	16 (4.9)	0 (0)
FOLFOX + avastin	1 (0.3)	0 (0)	0 (0)
Oxaliplatin alone	3 (0.9)	2 (0.6)	0 (0)
Age in range (years)			
30–50	37 (14.5)	8 (2.5)	0 (0)
51–60	44 (13.7)	7 (2.2)	0 (0)
61–78	146 (45.3)	38 (11.8)	4 (1.2)
79–88	23 (7.1)	14 (4.3)	1 (0.3)
Previous chemotherapy			
No	235 (72.9)	62 (19.3)	5 (1.5)
Yes	15 (4.7)	5 (15.6)	0 (0)
Cancer			
Adjuvant	181 (56)	48 (14.9)	4 (1.2)
Metastatic	69 (21.4)	19 (5.9)	1 (0.3)

## Data Availability

The data used to support the findings of this study are available from the corresponding author upon request.
